# The role of micronutrients and serum metabolites in intervertebral disk degeneration: insights from a Mendelian randomization study and mediation analysis

**DOI:** 10.3389/fnut.2024.1428403

**Published:** 2024-10-21

**Authors:** Nizhou Jiang, Quanxiang Wang, Jian Jiang, Lei Li

**Affiliations:** ^1^Department of Spine Surgery, Central Hospital of Dalian University of Technology, Dalian, China; ^2^Department of Spine Surgery, The First Affiliated Hospital of Dalian Medical University, Dalian, China; ^3^Department of Otolaryngology-Head and Neck Surgery, The Second Affiliated Hospital of Dalian Medical University, Dalian, China; ^4^Department of Orthopaedic Surgery, Shengjing Hospital of China Medical University, Shenyang, China

**Keywords:** intervertebral disk degeneration, Mendelian randomization, mediated Mendelian randomization, micronutrients, serum metabolites, vitamin B12

## Abstract

**Background:**

Intervertebral disk degeneration (IVDD) is a complex degenerative skeletal condition, potentially influenced by micronutrients and serum metabolites in its etiology. However, the exact causal relationship between these factors and IVDD remains ambiguous.

**Methods:**

The research employed a Two-Sample Mendelian Randomization (2SMR) analysis to thoroughly evaluate the causal relationship between 15 micronutrients (consisting of 7 minerals and 8 vitamins) as exposure variables, 1,091 blood metabolites, and 309 metabolite ratios as intermediary factors, and IVDD as the outcome. Additionally, reverse MR analysis and mediation analysis were carried out to validate the reliability of the results and explore the underlying mechanism by which micronutrients influence the risk of IVDD by regulating metabolites.

**Results:**

Among the micronutrients examined, vitamin B12 exhibited a noteworthy negative correlation with the incidence of IVDD (OR: 0.752, 95% [CI]: 0.573–0.987, *p* = 0.040), indicating a potential reduction in IVDD risk with increased vitamin B12 consumption. Of the 1,091 blood metabolites and 309 metabolite ratios analyzed, 52 metabolites displayed significant associations with IVDD, primarily linked to amino acid, fatty acid, nucleotide, and sphingolipid metabolic pathways. Mediation analysis identified 4-acetaminophen sulfate as a potential mediator in the protective effect of vitamin B12 against IVDD.

**Conclusion:**

This study has shown that vitamin B12 may reduce the risk of IVDD and has identified 52 serum metabolites that are associated with IVDD. Furthermore, it proposes that 4-acetaminophen sulfate could serve as a potential mechanism by which vitamin B12 exerts its inhibitory effects on IVDD.

## 1 Introduction

Intervertebral disk degeneration (IVDD) is a notable factor in the morbidity of the elderly population, significantly impacting quality of life through the presence of chronic back pain and limitations in mobility. This condition is distinguished by a progression of degenerative alterations in the structure of the disk, encompassing inflammatory mechanisms, apoptosis of disk cells, and degradation of the extracellular matrix (1). These pathological changes result in desiccation of the nucleus pulposus, fissures in the annulus fibrosus, and diminished disk height (2). Various treatment options exist, including conservative management and surgical intervention, but there is a noticeable absence of effective early prevention methods. Recent epidemiological findings indicate a possible connection between micronutrient imbalances, metabolic disturbances, and the development of skeletal disorders like IVDD (3, 4). This highlights the necessity of exploring the impact of specific micronutrients and serum metabolites on the pathogenesis of disk degeneration (5), potentially leading to innovative nutritional interventions and preventive measures.

Recent studies have shown a growing interest in exploring the correlation between micronutrient consumption and IVDD. Research has indicated a significant association between specific micronutrients, such as vitamins D, C, K, and zinc, and susceptibility to IVDD. For instance, a lack of 1,25-dihydroxyvitamin D (1,25(OH)2D) has been found to hasten the progression of IVDD by impeding the synthesis of extracellular matrix proteins and facilitating their breakdown (6). Furthermore, vitamin K2 seems to regulate inflammatory responses in IVDD by controlling the expression of Socs3 and Hmox1 genes (7). Zinc ions have been implicated in the regulation of matrix metalloproteinase activity in intervertebral disk cells, indicating that alterations in intracellular zinc levels may have a significant impact on the development of IVDD (8). Additionally, the FokI polymorphism within the vitamin D receptor (VDR) gene has been shown to modulate the response of intervertebral disk cells to vitamin D; specifically, cells with the FF genotype exhibit increased synthesis of matrix proteins and reduced expression of degrading enzymes in response to vitamin D, in contrast to those with the Ff genotype (9). This discovery offers novel insights into the potential advantages of vitamin D supplementation for individuals with IVDD. Mechanical overload is acknowledged as a risk factor for IVDD. Recent research indicates that selenium supplementation may mitigate ferroptosis in disk cells caused by mechanical stress by activating the Se-GPX4 and Se-SelK pathways, thus preserving extracellular matrix integrity (10). However, research on the association between other micronutrients and IVDD remains limited. Recent studies have also focused on the role of serum metabolites in IVDD. Notable fluctuations in metabolite concentrations have been observed in individuals with IVDD, suggesting a metabolic component in the disease’s pathogenesis. For instance, a study conducted by Wang et al. (4) demonstrated that a high-fat diet not only improved symptoms of IVDD in rats but also positively altered their serum metabolite profiles, underscoring the potential impact of metabolic alterations on IVDD. Moreover, Ji et al. (11) performed a Mendelian randomization (MR) analysis on a cohort of 157 Nordic individuals, revealing robust correlations between 13 serum metabolites, specifically within lipid and amino acid metabolic pathways, and IVDD. Despite these findings, further investigation into the relationship between serum metabolites and IVDD is in its nascent stages, highlighting a notable deficiency in thorough, prospective metabolomic research.

MR analysis utilizes genetic variations as instrumental variables to mimic the structure of conventional randomized controlled trials, thereby mitigating the impact of confounding variables (12). The increased prevalence of genome-wide association studies (GWAS) and the larger sample sizes in GWAS meta-analyses have bolstered the credibility of MR analysis in pinpointing risk factors associated with intricate diseases. Furthermore, the emergence of high-quality genetic instrumental variables derived from GWAS summary data on micronutrients and metabolites has broadened the applicability of MR analysis (13). Furthermore, mediation analysis offers a rigorous quantitative technique for assessing the impact of mediators on the causal pathways linking exposure variables and outcomes, grounded in principles of causal inference. This methodology is essential for elucidating the underlying mechanisms of causal associations (14). Consequently, the present study will employ a two-sample Mendelian randomization (2SMR) approach, leveraging recent GWAS data on micronutrients, serum metabolites, and IVDD. The aim is to examine the causal relationships between micronutrients and IVDD, as well as to investigate the potential mediating effects of metabolites through MR mediation analysis.

## 2 Methods

### 2.1 Data sources

**Figure 1:**
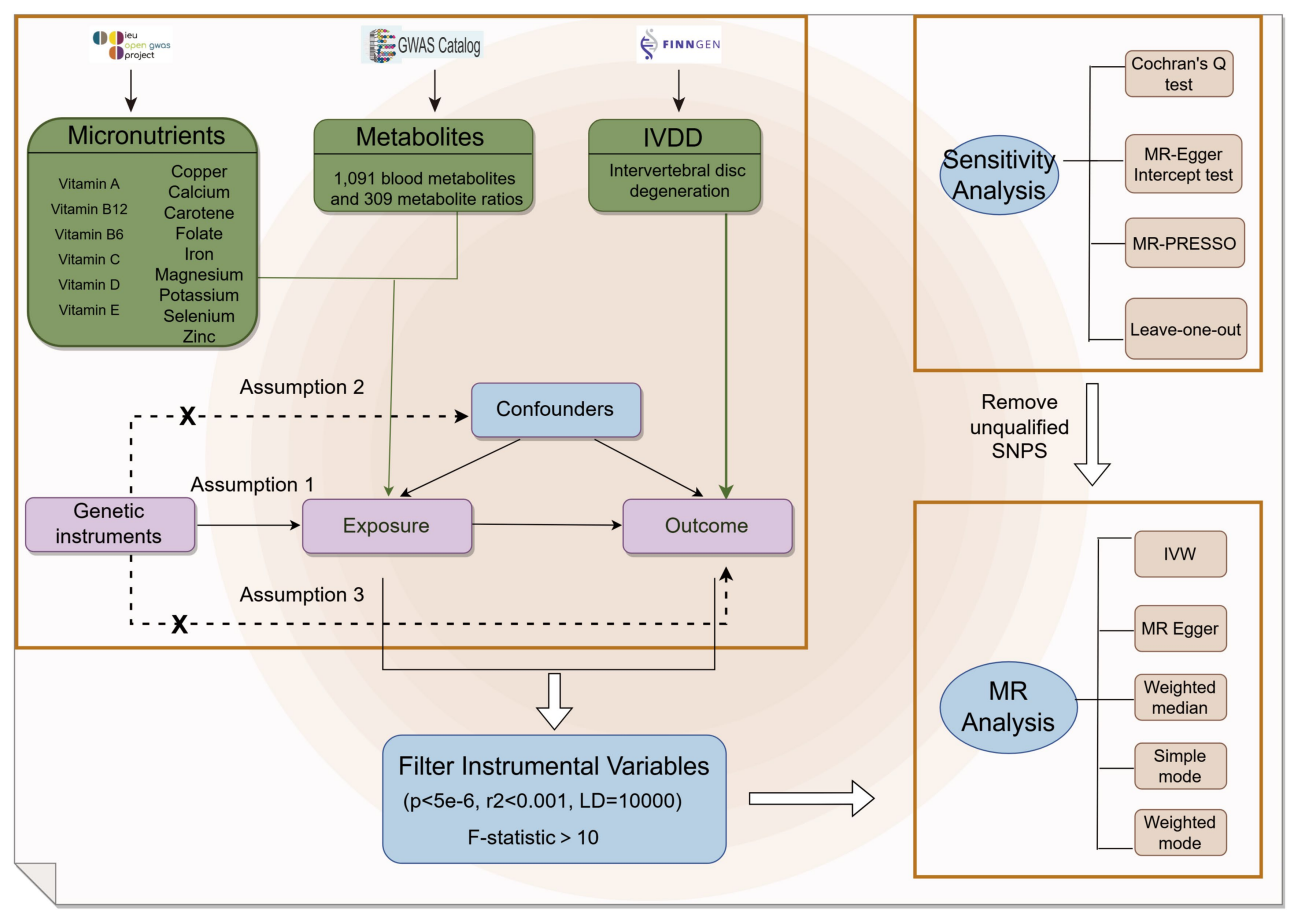
Flow chart of the study. Mendelian randomization study rationale: assumption 1, genetic instruments are associated with exposure; assumption 2, genetic instruments are not associated with confounders; assumption 3, genetic instruments are not associated with outcome, and genetic instruments act on outcome only through exposure.

This research examined summary data for 15 micronutrients, consisting of seven minerals and eight vitamins, obtained from the GWAS database^1^ as outlined in Table 1. Furthermore, we integrated genome-wide association findings for 1,091 blood metabolites and 309 metabolite ratios as reported by Zhao et al. (13). In order to validate the genetic instrumental variables, stringent selection criteria were employed. Only loci meeting the following criteria were included: (1) demonstrating genome-wide significance (*p* < 5 × 10^−6^) in GWAS; (2) exhibiting low linkage disequilibrium (LD) with an *r*^2^ < 0.001, and spatially separated by more than 10,000 kb; and (3) possessing an F-statistic exceeding 10, indicating strong instrument strength, calculated as F = R^2^(*n*−2)/(1-*R*^2^), where n represents the effective sample size for single nucleotide polymorphisms (SNPs) association analysis in GWAS and R^2^ represents the explained phenotypic variance by the instrumental variable (15). The GWAS summary data for IVDD, identified as “finn-b-M13_INTERVERTEB,” were acquired from the FinnGen consortium,^2^ which included 20,001 cases and 164,682 controls (Figure 1).

**Table 1:** The detailed information on the GWAS of micronutrients.

Micronutrients	Sample size	Population	GWAS ID
Copper	2,603	European	ieu-a-1073
Calcium	64,979	European	ukb-b-8951
Carotene	64,979	European	ukb-b-16202
Folate	64,979	European	ukb-b-11349
Iron	64,979	European	ukb-b-20447
Magnesium	64,979	European	ukb-b-7372
Potassium	64,979	European	ukb-b-17881
Selenium	2,603	European	ieu-a-1077
Zinc	2,603	European	ieu-a-1079
Vitamin A	460,351	European	ukb-b-9596
Vitamin B12	64,979	European	ukb-b-19524
Vitamin B6	64,979	European	ukb-b-7864
Vitamin C	64,979	European	ukb-b-19390
Vitamin D	64,979	European	ukb-b-18593
Vitamin E	64,979	European	ukb-b-6888

### 2.2 MR analysis

In this research, we utilized the 2SMR method with summary-level data from GWAS to investigate potential causal associations between 15 micronutrients and IVDD (16). Our selection of SNPs as genetic instrumental variables for each micronutrient was based on rigorous criteria, with these SNPs acting as instrumental variables for the exposure and IVDD serving as the outcome variable. Univariate 2SMR analyses were performed for each micronutrient using the “TwoSampleMR” package (17) in R (version 4.1.3), with a primary focus on employing the Inverse Variance Weighted (IVW) method to estimate causal effects. The robustness of our results was additionally confirmed through various sensitivity analyses, such as MR-Egger regression, the weighted median method, simple mode, and weighted mode approaches, as well as Cochrane’s Q test to evaluate heterogeneity among the instrumental variables for each micronutrient (18–20). Further sensitivity analyses were performed to assess the potential impact of horizontal pleiotropy, utilizing the MR-Egger intercept test, the Mendelian Randomization Pleiotropy RESidual Sum and Outlier (MR-PRESSO) global test (21), and Leave-One-Out Analysis. These assessments were designed to identify and correct for any pleiotropic influences that may introduce bias into the causal inferences. All statistical tests were carried out using a significance threshold of *p* < 0.05 to ensure the reliability of our results.

### 2.3 Reverse MR analysis

In order to explore the potential mediating effect of serum metabolites on the relationship between trace elements and IVDD, we performed a reverse MR analysis on trace elements that did not show a reverse causal relationship with IVDD in an initial MR evaluation of 15 trace elements. This analysis was conducted to eliminate the possibility of reverse causality between trace elements and IVDD. We employed the IVW method as our principal statistical technique, supplemented by various sensitivity analyses such as MR-Egger regression, the weighted median method, simple mode, and weighted mode methods to thoroughly evaluate the robustness of the reverse causal relationships. Within the framework of reverse MR, IVDD was considered as the exposure variable, while the concentrations of trace elements were regarded as the outcome variable. Through the integration of results from various methodologies, we pinpointed trace elements that did not exhibit substantial reverse causal relationships with IVDD. This specific selection allowed for a more in-depth examination of the mediating influences of serum metabolites in subsequent mediation MR analyses. A significance threshold of *p* < 0.05 was consistently upheld for all statistical analyses.

### 2.4 Mediation analysis

In order to investigate potential serum metabolite mediators in the confirmed positive correlations between trace elements and IVDD, a two-step mediation analysis approach was employed. Initially, univariable 2SMR analysis was carried out utilizing the “TwoSampleMR” package. This analysis employed screened serum metabolite SNPs as exposures and IVDD as the outcome. The IVW method was selected as the primary method to estimate causal effects, and was supported by sensitivity analyses, including MR-Egger regression, the weighted median method, simple mode, and weighted mode methods. Additionally, heterogeneity among genetic instrumental variables was assessed using Cochrane’s Q test. The influence of horizontal pleiotropy and outliers was evaluated using the MR-Egger intercept test, MR-PRESSO global test, and leave-one-out analysis to ensure the robustness and validity of our findings.

In the second phase of our analysis, serum metabolites demonstrating significant causal effects from the initial analysis were designated as outcome variables, while trace elements exhibiting a unidirectional causal relationship with IVDD risk were used as exposure variables. We conducted a univariable 2SMR analysis using the IVW method to identify potential mediators. For estimating the effect size of each mediator, we employed the product of coefficients method (22). This approach first calculates the effect value beta1 of trace elements on mediators, then determines the adjusted effect value beta 2 of mediators on IVDD. The product of beta1 and beta2 yields the indirect effect, representing the influence of trace elements on IVDD through mediators (23). To uphold a fundamental assumption of mediation MR analysis—that the genetic instrumental variables for estimating beta1 and beta2 should be independent—we excluded SNPs significantly influenced by trace elements in the beta2 estimation to prevent redundancy. The proportion of the total effect of trace elements on IVDD accounted for by each mediator is quantified by dividing the indirect effect by the total effect. Standard errors were calculated using the delta method, drawing on the effect estimates from the 2SMR analysis.

## 3 Results

### 3.1 MR analysis of micronutrients and IVDD

**Figure 2:**
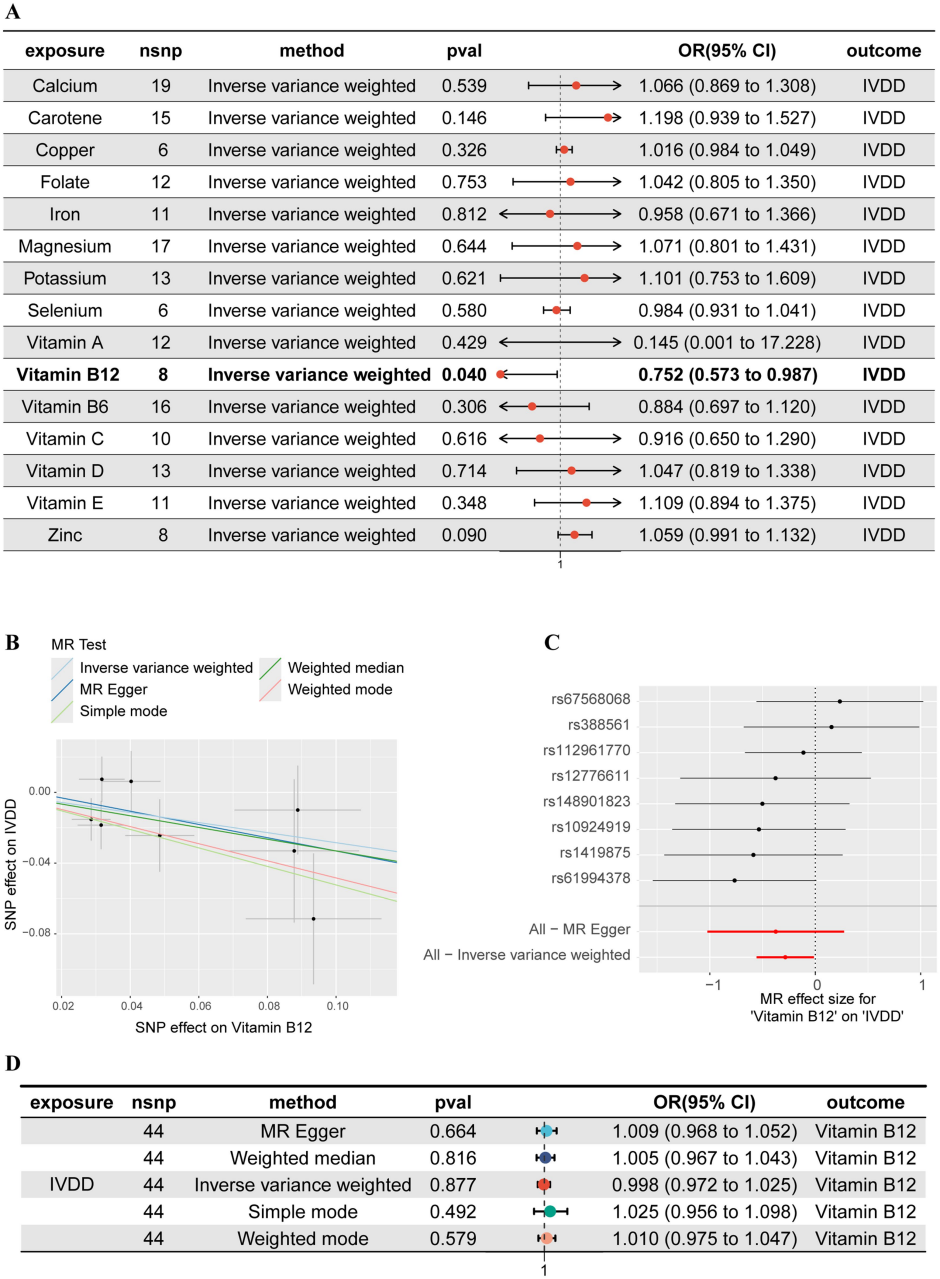
Mendelian randomization (MR) and reverse MR analyses of 15 micronutrients and IVDD. **(A)** Forest plot of the MR analyses between 15 micronutrients and IVDD. **(B)** Scatter plot of the MR analysis between vitamin B12 and IVDD. **(C)** Forest plot of the MR analysis between vitamin B12 and IVDD. **(D)** Forest plot of the reverse MR analyses between 15 micronutrients and IVDD.

A 2SMR analysis was utilized to examine the association between micronutrients and IVDD, with 15 micronutrients being categorized into two groups consisting of 7 minerals and 8 vitamins (Figure 2A). The findings indicated that, of all the micronutrients examined, only vitamin B12 exhibited a statistically significant negative correlation with the likelihood of developing IVDD (OR: 0.752, 95%CI: 0.573–0.987, *p* = 0.040). These results suggest that augmenting one’s intake of vitamin B12 may potentially decrease the risk of IVDD (Figures 2B,C). The Cochrane’s Q test results (*Q* = 5.710, *p* = 0.573) revealed no significant heterogeneity among the instrumental variables used in the analysis. Additionally, the MR-Egger intercept test (egger_intercept = 0.0043, *p* = 0.773) and MR-PRESSO (*p* = 0.598) detected no horizontal pleiotropy, affirming the reliability of the MR findings in this study.

In order to address the potential for reverse causation between vitamin B12 and IVDD, we conducted a reverse MR analysis. Our analysis demonstrated that when considering IVDD as the exposure and vitamin B12 as the outcome, there was no significant causal relationship (Odds Ratio: 0.997, 95% Confidence Interval: 0.971–1.024, *p* = 0.877) (Figure 2D). These findings substantiate a unidirectional causal relationship from vitamin B12 to IVDD, unaffected by reverse causality, thus providing a robust basis for further mediation analysis.

### 3.2 MR analysis of serum metabolites and IVDD

**Figure 3:**
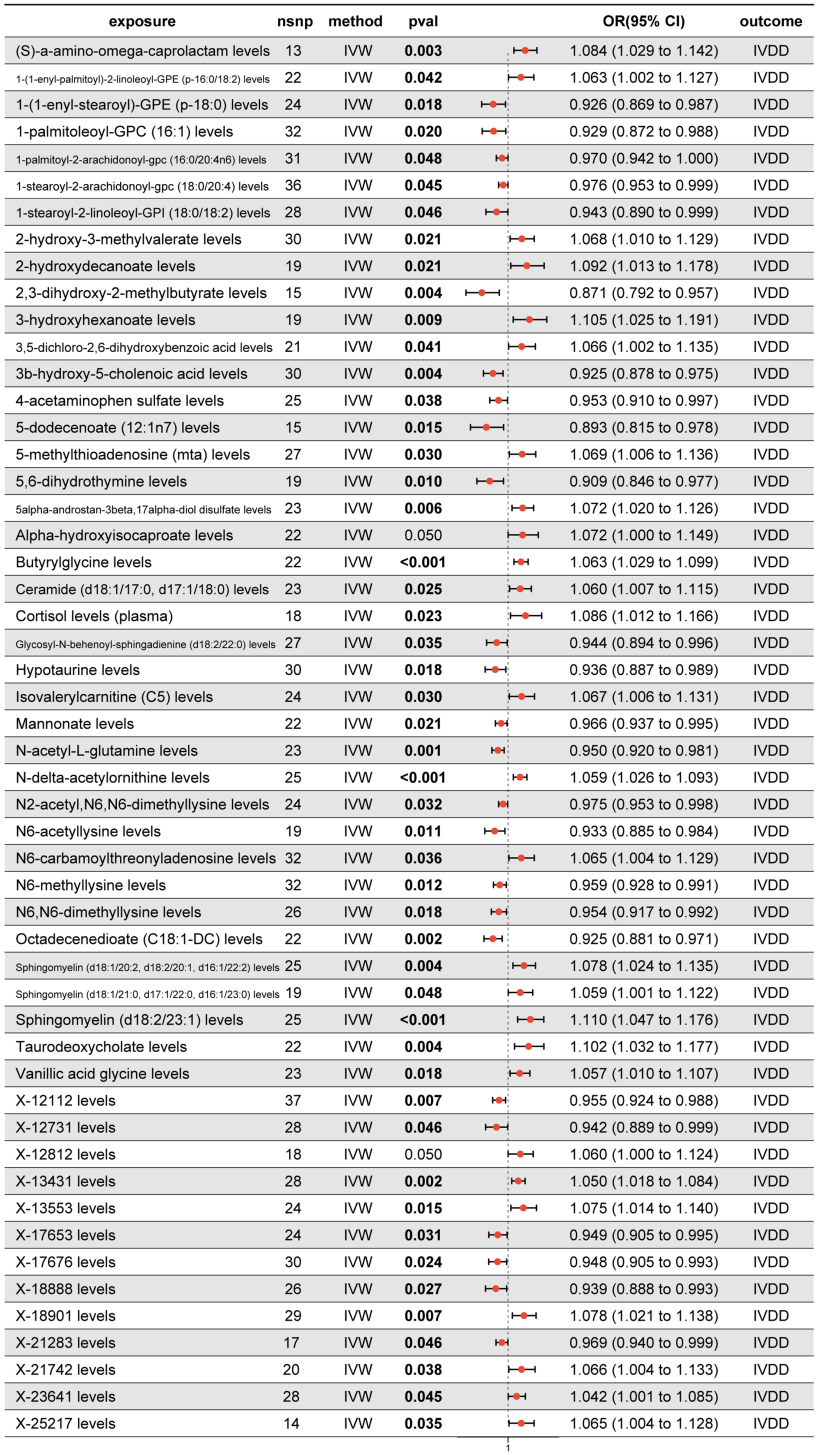
Forest plot illustrating the positive results from the MR analyses between 1,091 blood metabolites, 309 metabolite ratios, and IVDD.

To elucidate the causal relationship between blood metabolites and IVDD, we systematically analyzed 1,091 blood metabolites and 309 metabolite ratios employing a 2SMR approach. Prior to the analysis, genetic loci with linkage disequilibrium and weak instrumental variables were excluded to preserve the integrity of the study. Each metabolite then underwent univariable two-sample MR analysis, followed by tests for pleiotropy to identify metabolites with robust associations. Finally, we identified 52 serum metabolites significantly associated with IVDD (Figure 3). These include seven amino acid derivatives (S-*α*-amino-*ω*-caprolactam, 2-hydroxy-3-methylvalerate, alpha-hydroxyisocaproate, butyrylglycine, N-acetyl-L-glutamine, N-delta-acetylornithine, vanillic acid glycine); 14 fatty acid derivatives (1-(1-enyl-palmitoyl)-2-linoleoyl-GPE [p-16:0/18:2], 1-(1-enyl-stearoyl)-GPE [p-18:0], 1-palmitoleoyl-GPC [16:1], 1-palmitoyl-2-arachidonoyl-GPC [16:0/20:4n6], 1-stearoyl-2-arachidonoyl-GPC [18:0/20:4], 1-stearoyl-2-linoleoyl-GPI [18:0/18:2], 2-hydroxydecanoate, 3-hydroxyhexanoate, 3β-hydroxy-5-cholenoic acid, 5-dodecenoate [12,1n7], ceramide [d18:1/17:0, d17:1/18:0], glycosyl-N-behenoyl-sphingadienine [d18:2/22:0], octadecenedioate [C18:1-DC], taurodeoxycholate); five nucleotide derivatives (5-methylthioadenosine [MTA], 5,6-dihydrothymine, N6-carbamoylthreonyladenosine, N2-acetyl, N6,N6-dimethyllysine, N6,N6-dimethyllysine); four sphingolipids (sphingomyelin [d18:1/20:2, d18:2/20:1, d16:1/22:2], sphingomyelin [d18:1/21:0, d17:1/22:0, d16:1/23:0], sphingomyelin [d18:2/23:1], isovalerylcarnitine [C5]); seven other known metabolites (5α-androstan-3β,17α-diol disulfate, cortisol, 2,3-dihydroxy-2-methylbutyrate, 3,5-dichloro-2,6-dihydroxybenzoic acid, 4-acetaminophen sulfate, hypotaurine, mannonate); and 15 unknown metabolites. These findings provide valuable insights into the metabolic pathways potentially contributing to IVDD.

### 3.3 Mediation analysis of 52 serum metabolites in the causal relationship between vitamin B12 and IVDD

**Figure 4:**
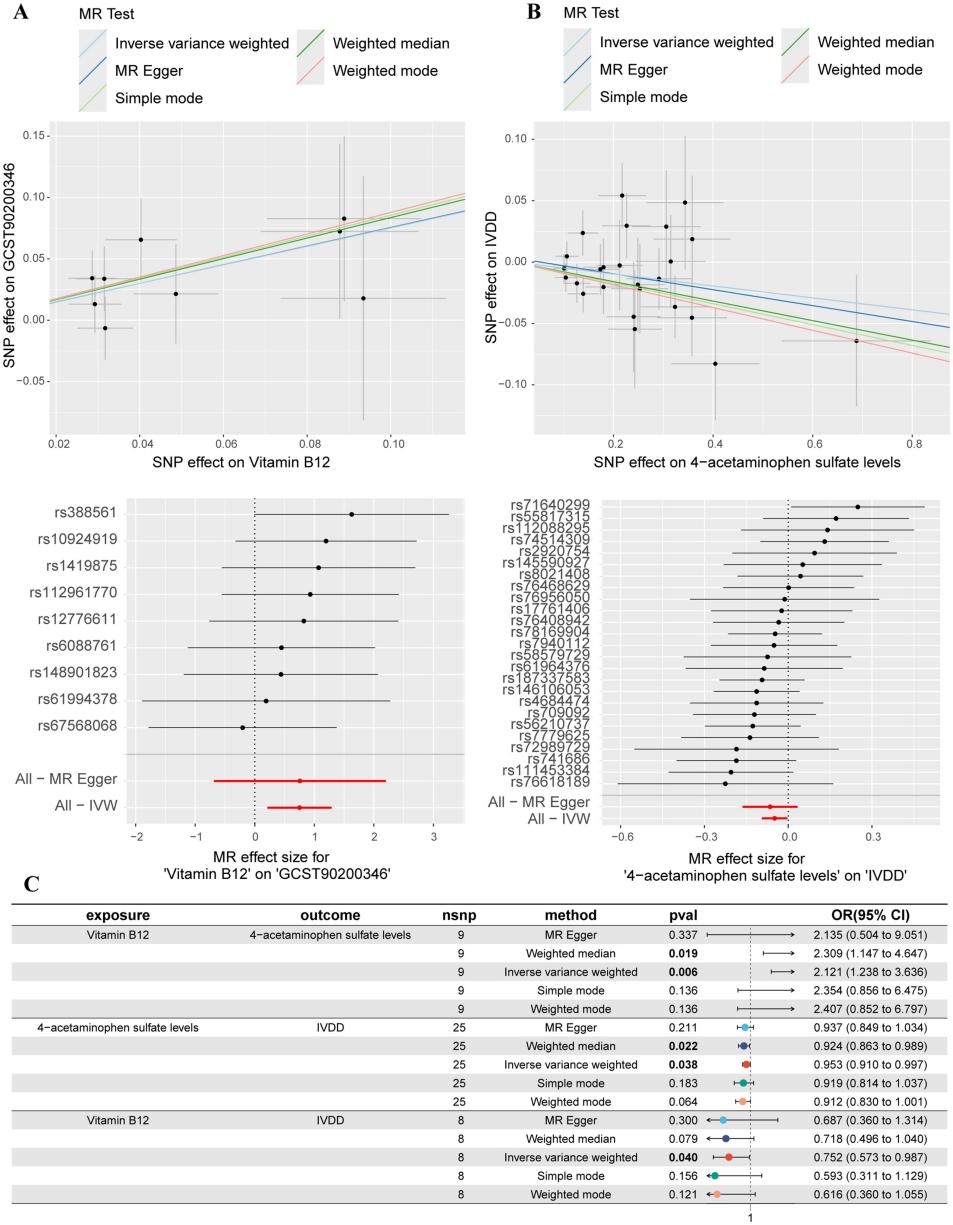
Mediation MR analyses. **(A)** Scatter plot and forest plot of the MR analysis between vitamin B12 and 4-acetaminophen sulfate levels. **(B)** Scatter plot and forest plot of the MR analysis between 4-acetaminophen sulfate levels and IVDD. **(C)** Summary forest plot of the mediation analyses.

**Figure 5:**
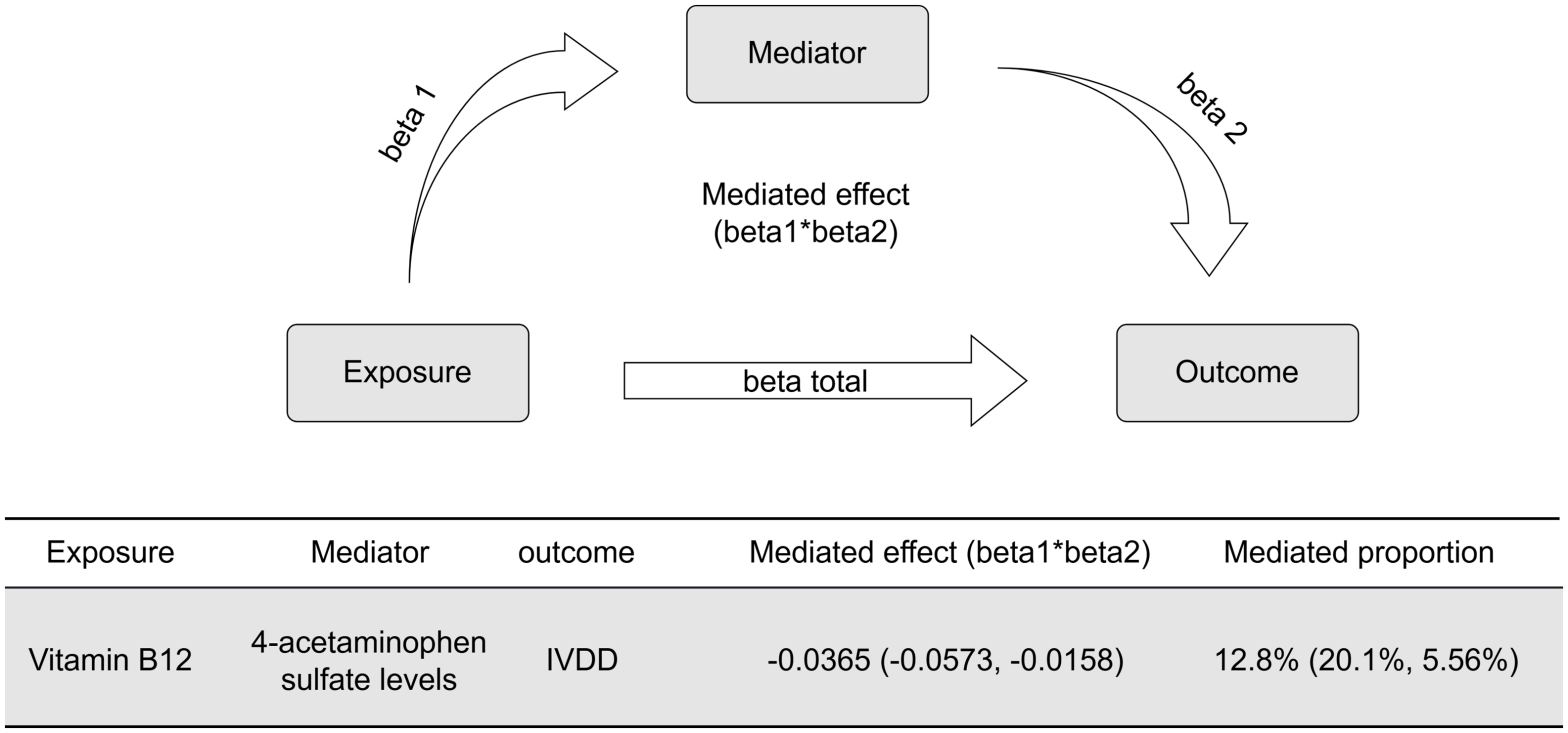
Mediation effects and proportion mediated by serum metabolites.

In order to explore the potential mediating effect of serum metabolites on the causal association between vitamin B12 and IVDD, we conducted a systematic mediation analysis using a two-step methodology. Initially, 2SMR analyses were performed with vitamin B12 as the exposure and 52 IVDD-associated serum metabolites as outcomes to estimate the effect size (beta1). Among these metabolites, only 4-acetaminophen sulfate demonstrated a significant causal relationship with vitamin B12 (OR: 2.121, 95% CI: 1.237–3.636, *p* = 0.006, beta1 = 0.752) (Figure 4A). The results of the Cochrane’s Q test (*Q* = 3.609, *p* = 0.890) indicated an absence of significant heterogeneity across the instrumental variables utilized in the analysis. Further, both the MR-Egger intercept test (egger_intercept = −0.00027, *p* = 0.992) and MR-PRESSO (*p* = 0.915) showed no evidence of horizontal pleiotropy. In the second step, excluding significant genetic instrumental variables involved in calculating beta1, we assessed the effect of 4-acetaminophen sulfate on IVDD (beta2). This analysis revealed that increased levels of 4-acetaminophen sulfate significantly reduced the risk of IVDD (OR: 0.952, 95% CI: 0.909–0.997, *p* = 0.038, beta2 = −0.048) (Figure 4B). The Cochrane’s Q test (*Q* = 22.659, *p* = 0.539) showed no significant heterogeneity among the instrumental variables. Additionally, the MR-Egger intercept test (egger_intercept = 0.00343, *p* = 0.719) and MR-PRESSO (*p* = 0.547) found no horizontal pleiotropy, confirming the reliability of the MR results in this study. To determine the total effect of vitamin B12 on IVDD, a direct MR analysis was performed, showing a significant reduction in IVDD risk with increased vitamin B12 levels (OR: 0.752, 95% CI: 0.573–0.987, *p* = 0.040, beta total = −0.284) (Figure 4C). Finally, we incorporated beta1, beta2, and beta total into the mediation model to calculate the mediated effect of 4-acetaminophen sulfate. Our findings suggest that vitamin B12 may reduce the risk of IVDD by promoting increased levels of 4-acetaminophen sulfate [Mediated effect (beta1*beta2): −0.0365 (−0.0573, −0.0158)] [Mediated proportion: 12.8% (20.1, 5.56%)] (Figure 5).

## 4 Discussion

The purpose of this study was to identify micronutrients that may reduce the risk of IVDD and to elucidate the serum metabolites mediating this protective effect. We employed several analytic methods, including 2SMR, reverse MR, and mediation MR. Our results suggest that vitamin B12 could decrease the risk of IVDD by enhancing the expression of the serum metabolite 4-acetaminophen sulfate.

The correlation between micronutrients and IVDD is becoming more acknowledged in the fields of orthopedics and nutrition. Micronutrients play a critical role in numerous metabolic processes and physiological functions, aiding in the maintenance of the structural and functional integrity of connective tissues like bone and cartilage (24). Therefore, the examination of how micronutrients influence IVDD is essential for the advancement of nutritional interventions and preventative measures. The present study examined 15 micronutrients, comprising 7 minerals and 8 vitamins, with the objective of elucidating their potential causal relationships with susceptibility to IVDD. Previous research has underscored the correlation between zinc and the advancement of IVDD, wherein elevated zinc concentrations in disk cells trigger the activation of matrix metalloproteinases (MMPs), thereby hastening the degradation of the extracellular matrix. Conversely, diminished zinc levels under hypoxic conditions may impede MMP function, suggesting a plausible protective mechanism against cellular injury (8). Selenium, a cofactor essential for the activity of antioxidant enzymes like glutathione peroxidase (GPX4), is pivotal in attenuating ferroptosis and oxidative stress in intervertebral disk cells. Research indicates that mechanical stress can trigger Piezo1 ion channels, resulting in calcium overload and endoplasmic reticulum stress in these cells, thereby exacerbating ferroptosis. Supplementation with selenium has been demonstrated to decrease intracellular free calcium levels and ameliorate oxidative stress and ferroptosis, consequently reducing the likelihood of IVDD. These results emphasize the considerable protective effects of micronutrients in preventing disk degeneration (10, 25). However, the MR analysis did not find a causal relationship between the seven minerals examined and the risk of IVDD. This lack of association may be due to constraints related to the genetic instrumental variables utilized and the limited sample size.

The results of our study demonstrate a notable causal association between vitamin B12 and IVDD, suggesting a potentially more pronounced connection with vitamins compared to minerals. Epidemiological research has established a robust correlation between vitamin D insufficiency and degeneration of the lumbar disks, with evidence indicating that vitamin D supplementation effectively reduces this risk (26). *In vitro* investigations indicate that vitamin D promotes the integrity of nucleus pulposus cells by increasing Sirt1 expression, suppressing the NF-κB inflammatory pathway, and decreasing MMPs and inflammatory mediators (6). The depletion of VDR has been shown to markedly diminish cell proliferation and matrix synthesis, hastening apoptosis and senescence. Nevertheless, administration of vitamin D has been found to partially counteract these deteriorative effects by activating Sirt1 and inhibiting the NF-κB pathway (9, 27). Furthermore, the deficiency of vitamin C, frequently seen alongside inadequate levels of vitamin D, poses a potential risk factor for IVDD in the elderly population. Vitamin C plays a crucial role in collagen synthesis, and its insufficiency can impair the functionality of connective tissues by diminishing collagen production and compromising structural integrity. Additionally, the progression of IVDD may lead to localized tissue inflammation, which can exacerbate the depletion of vitamin C levels, perpetuating a detrimental cycle that accelerates disk degeneration (28).

Furthermore, alongside vitamins D and C, vitamin B12 is being recognized as a potentially significant factor in the development of IVDD. While direct evidence linking vitamin B12 to IVDD is currently limited, several studies suggest that vitamin B12 may offer protection against IVDD through mechanisms involving anti-inflammatory, antioxidative, and autophagy-regulating pathways. Inflammation and oxidative stress play crucial roles in the pathogenesis of IVDD. Experimental models have shown that supplementation with vitamin B12 can reduce levels of inflammatory mediators such as IL-1β and IL-6, as well as decrease malondialdehyde (MDA) levels and enhance glutathione content. Furthermore, vitamin B12 has been shown to suppress the TLR-4/NF-κB signaling pathway, leading to the inhibition of subsequent inflammatory reactions (29). A study also demonstrated that supplementation with a B-vitamin complex, which includes folic acid, vitamin B6, and B12, improved neurobehavioral deficits in offspring mice exposed to maternal PM2.5 by regulating neuroinflammatory markers and decreasing oxidative stress (30). Studies in diabetic rat models have also demonstrated that vitamin B12, combined with folic acid, alleviates nicotine-induced insulin resistance and pancreatic *β*-cell apoptosis through antioxidative and anti-inflammatory actions (31). Moreover, research has indicated that vitamin B12 can provide protective benefits against tissue damage by regulating autophagy. High doses of vitamin B12 have been found to effectively decrease oxidative stress and apoptosis in cases of myocardial ischemia–reperfusion injuries by increasing SIRT3 and AMPK activity, inhibiting Nox2 expression, and reducing levels of cleaved caspase-3 (32). *In vitro*, vitamin B12 has also shown to safeguard RIN-m5F pancreatic β-cells from high glucose-induced apoptosis by activating autophagy (33). Moreover, vitamin B12 was found to reduce infarct volume in a rat model of focal cerebral ischemia by upregulating the ERK1/2 pathway and Beclin1 expression, while downregulating Bax and cleaved caspase-3 (34). Collectively, these results collectively indicate that vitamin B12 may provide protective effects in conditions like IVDD by reducing inflammation, alleviating oxidative stress, and regulating autophagy.

In addition to its known anti-inflammatory, antioxidative, and autophagy-regulating effects, vitamin B12 may play a role in the development and progression of IVDD by influencing metabolite levels in the body. Using 2SMR, we investigated the potential causal relationships between 1,091 blood metabolites and 309 metabolite ratios with IVDD. Our analysis revealed 52 serum metabolites that were significantly linked to IVDD, including amino acid derivatives, fatty acids, nucleotides, sphingosine, and sphingolipids. Prior research supports the association between the progression of IVDD and notable metabolic changes that can impact the development of the condition by influencing multiple pathological mechanisms, including inflammation, apoptosis, autophagy, and matrix degradation in disk cells. Specifically, the accumulation of lactate, a byproduct of glycolysis, in degenerated disks has been identified as a potential inducer of senescence and oxidative stress in nucleus pulposus cells, potentially through the modulation of the PI3K/Akt pathway (35). Furthermore, metabolomic investigations have revealed disturbances in lipid metabolism, particularly involving phospholipids, cholesterol esters, and triglycerides, as well as in bile acid metabolism among individuals with IVDD. These findings indicate the potential of these metabolites as biomarkers and targets for therapeutic intervention (4, 11). The metabolites identified in this study, including various fatty acids (e.g., 2-hydroxydecanoate, octadecenedioate) and sphingolipids (e.g., sphingomyelin), align with prior metabolomic findings (36). Furthermore, certain metabolites, including 5′-methylthioadenosine (MTA), a sulfur-containing nucleoside, have been observed in other musculoskeletal disorders and may impact IVDD through comparable mechanisms. Recent studies have shown that MTA has the potential to alleviate inflammation-induced bone loss by inhibiting the RANKL-induced NFκB-NFATc1 signaling pathway and suppressing osteoclast differentiation and activity, thus regulating inflammatory reactions in IVDD (37).

The results of further mediation analysis suggest that heightened levels of vitamin B12 may have a significant impact on reducing the risk of IVDD, potentially through the mediation of increased levels of 4-acetaminophen sulfate. Vitamin B12, an essential coenzyme involved in the methionine cycle, plays a crucial role in the regulation of homocysteine metabolism and the maintenance of sulfate homeostasis within the body (38). This investigation highlights the ability of vitamin B12 to substantially raise levels of 4-acetaminophen sulfate, potentially by enhancing the methionine cycle, increasing sulfate availability, and preserving sulfotransferase function. Moreover, previous studies have indicated that vitamin B12 can improve liver detoxification functions by enhancing acetaminophen sulfation metabolism (39). This process may lead to the inhibition of IVDD progression through the modulation of inflammatory responses, regulation of matrix metabolism, and protection of intervertebral disk cell function. Additionally, elevated levels of the sulfated metabolite, 4-acetaminophen sulfate, suggest an increased detoxification capacity, which may aid in the efficient clearance of toxic substances related to IVDD and reduce their detrimental impact on intervertebral disks (40).

Although this study employed MR analysis to investigate the causal relationships between micronutrients, metabolites, and IVDD, several limitations exist. First, selection bias and the limited strength of genetic instrumental variables may affect the analytical power. Second, due to gaps in current metabolomic and nutriomic studies, many micronutrients and metabolites lack suitable genetic instruments and were excluded, potentially underestimating their impact on IVDD. Additionally, we did not apply multiple testing corrections such as Bonferroni adjustment, as our primary objective was to identify potential biomarkers or therapeutic targets for IVDD. The stringent Bonferroni criteria might exclude meaningful indicators. Furthermore, while mediation analysis suggests a mediating effect of 4-acetaminophen sulfate in the association between vitamin B12 and IVDD, the precise molecular mechanisms remain unclear. The effects of vitamin B12 may involve various pathological pathways not comprehensively explored in this study. Future research should expand sample sizes, include diverse populations, conduct prospective cohort studies and randomized controlled trials, and incorporate histopathological assessments to further validate the causal relationships and underlying mechanisms.

## 5 Conclusion

This study utilized MR analysis to establish a significant association between vitamin B12 and a decreased risk of IVDD. Out of the 1,091 blood metabolites and 309 metabolite ratios analyzed, 52 were found to be correlated with IVDD risk. Particularly noteworthy is the potential role of 4-acetaminophen sulfate in mediating the protective effects of vitamin B12. This discovery unveils a new pathway by which vitamin B12 may reduce IVDD risk by influencing the metabolism of 4-acetaminophen sulfate, thereby enhancing our comprehension of the mechanisms involved in IVDD progression.

## Data Availability

The original contributions presented in the study are included in the article/supplementary material, further inquiries can be directed to the corresponding author.

## Footnotes

^1^ https://gwas.mrcieu.ac.uk

^2^ https://www.finngen.fi/en
